# Validation and Analysis of *COIL*, a Gene Associated with Multiple Lambing Traits in Sheep

**DOI:** 10.3390/genes15020235

**Published:** 2024-02-13

**Authors:** Hang Cao, Yilin Wen, Haiyu Ma, Wujun Liu

**Affiliations:** College of Animal Science, Xinjiang Agricultural University, Urumqi 830052, China; y77996699@126.com (H.C.); yilinwen@126.com (Y.W.); wujunliu1026@xjau.edu.cn (W.L.)

**Keywords:** *COIL* gene, single-nucleotide polymorphism, lambing number trait, association analysis

## Abstract

In a past study, the team used specific-locus amplified fragment sequencing (SLAF sequencing) to detect single-nucleotide polymorphisms (SNPs) contributing to the differences in lambing numbers in Xinjiang sheep. This study verified the correlation between the *COIL* gene and lambing number characters in sheep and explored its possible mechanism of action. In this study, three SNPs in the *COIL* gene, namely COILSNP1 (rs7321466), COILSNP2 (rs7314134), and COILSNP3 (rs7321563), were explored in terms of their possible mechanism of action. A tissue expression profiling analysis revealed that the *COIL* gene was significantly more expressed in the uterus and ovaries than in other tissues (*p* < 0.05), whereas an association analysis revealed that the number of lambs born was significantly different among individuals with different genotypes of this COILSNP1 (*p* < 0.05). The Cell Counting Kit-8(CCK-8) revealed that the overexpression of the *COIL* gene significantly increased the proliferation of mouse ovarian fibroblasts and sheep fibroblasts (*p* < 0.05). The 3-(4,5-dimethylthiazol-2-yl)-2,5-diphenyltetrazolium bromide (MTT) revealed that the overexpression of the *COIL* gene significantly increased the activity of sheep fibroblasts (*p* < 0.01) and mouse ovarian fibroblasts (*p* < 0.05). The overexpression of the *COIL* gene affected the biogenesis pathway of spliceosomal U snRNPs by validating protein network connections. This activity affects ovulation, embryonic development, and changes in lambing size in sheep.

## 1. Introduction

The destruction of natural grasslands in China and the market demand for sheep meat have paved the way for the intensive farming mode of production. The most crucial aspect of production under this strategy is the number of lambs produced [1]. Over 45% of the value of the livestock industry’s production is attributed to breeds, such that the production of breeds with high lambing rates has emerged as a priority [2]. The study of master-effective genes for sheep lambing number, a complex quantitative trait, is a popular topic in sheep breeding research [3]. Various patterns in quantitative traits are currently based on the micro-effective polygenic hypothesis, and the *FecB* gene, discovered in Australia, can only be used to identify the lambing number in individual breeds [4]. The discovery of these genes and markers will help China, a country with a huge sheep population, rapidly select new breeding populations of sheep with high lambing rates.

*COIL* is a characteristic protein of Cajal bodies, a conserved nucleolar organelle involved in multiple aspects of the biogenesis of small nuclear ribonucleoprotein (RNP). In plants and animals, *COIL* is required to maintain homeostasis within Cajal bodies [5]. *COIL* is required for the formation of Cajal bodies and recruitment of spliced small nuclear ribonucleoproteins (snRNPs), which are required to modify the guidance RNA and survival motor neuron (SMN) protein complexes. A knockout of *COIL* causes the residual Cajal bodies to lose contact with the SMN complex. Because the SMN is essential for the assembly and in vivo recycling of snRNPs. The lack of interaction between the SMN and *COIL* in the nucleus could lead to the reduced ability to assemble RNPs, causing downstream effects on the development and gametogenesis, thus affecting the lambing numbers. Research has shown that the effects of the removal of *COIL* on the overall viability and reproductive success in mice and found that *COIL* knockout mice significantly reduced the number of oocytes, lowered the production of oocytes capable of fertilization, and reduced the number of litters per litter, showing significant fertility and reproductive defects [6].

The lambing number groups of local sheep breeds from Xinjiang were subjected to specific-locus amplified fragment sequencing (SLAF sequencing) at an early stage. The genome-wide association analysis revealed that the sheep *COIL* gene was significantly linked to the lambing number [7]. To confirm the gene’s function, tissue expression validation, investigation of the pathways of occurrence operated upon, and association analysis of the *COIL* gene polymorphisms with lambing features were performed.

## 2. Materials and Methods

### 2.1. Animals and Sampling

A total of 427 Xinjiang local sheep production ewes (62 Hetan multiparous red sheep from Pishan county of Hetian city of Xinjiang, 65 Altay sheep from Fuhai county of Altay city of Xinjiang, 240 Turpan black sheep from Turpan city of Xinjiang, 44 Cele black sheep from Cele county of Xinjiang, and 16 Dorang sheep from Maigaiti county of Kashi city of Xinjiang) were used in *COIL* gene polymorphism detection. Sheep whole-blood samples were obtained and stored at −20 °C for genome extraction. Six Hetian multiparous red sheep were selected, at age one, as production ewes. A total of eight tissues, the heart, liver, kidneys, spleen, lungs, uterine horn, uterine body, and ovary, were collected after execution, snap-frozen in liquid nitrogen, and kept in the freezer at −80 °C to perform tissue expression. The Changping base of the Beijing Animal Husbandry and Veterinary Research Institute provided sheep fibroblasts, which were subsequently cultivated and passed through cell chambers. In addition, mouse ovarian fibroblasts were conserved for laboratory use.

### 2.2. Determination of Multi-Lambing Performance and Extraction of Tissue DNA in Sheep

The lambing number data were gathered to examine the average number of lambs using the sheep farm data statistics. The DNA from sheep whole blood was extracted using the standard phenol/chloroform procedure and backup samples were kept at −20 °C [8].

### 2.3. Polymorphism Detection of the COIL Gene in Sheep

The *COIL* gene on chromosome XI was tested for polymorphism (Table 1 for the primer sequences of the three loci of *COIL*). Bemec Biotechnology Co (Hangzhou, China). was contracted to genotype 472 sheep using the KASP typing method based on the three *COIL* gene single-nucleotide polymorphism (SNP) sites that were discovered during sequencing.

### 2.4. RNA Extraction, Reverse Transcription, and Real-Time PCR

The total RNA from sheep tissues or cells was extracted using the TRIzol reagent (Tiangen, Beijing, China). The quality of the collected RNA was verified using 1% agarose gel electrophoresis. According to the Promega ImProm-I (Promega, Madison, WI, USA) reverse transcription kit’s instructions, the extracted total RNA was reverse transcribed into cDNA using the following conditions: FastStart Universal SYBR Green Master (ROX) 5 μL, cDNA template 1 μL, top, bottom, and bottom, and real-time PCR was performed using FastStart Universal SYBR Green Master (Roche, Basel, Switzerland) reagents. Denaturation at 95 °C for 10 min, denaturation at 95 °C for 15 s, annealing at 60 °C for 30 s, and extension at 72 °C for 30 s were the reaction conditions. This involved a total of 40 cycles. The *GAPDH* gene was utilized as an internal reference, and the raw Ct measurements were transformed to relative gene expression with the 2^−ΔCt^ method using Ct = Ct _target gene_ −Ct _GAPDH_.

### 2.5. Construction and Western Blot Validation of the Eukaryotic Expression Vector of the Sheep COIL Gene

The full-length CDS region of the *COIL* gene amplification primers was designed using the sheep *COIL* gene sequence. The PCR amplification products were purified, recovered, and ligated with the pcDNA3.1-myc empty vector. Subsequently, the receptor cells were transformed, single colonies were picked, plasmids were extracted, and the sequences of the plasmids were subsequently determined. Sheep fibroblasts and mouse ovarian fibroblasts were transfected with the created pcDNA3.1-myc-COI using Lipofectamine 3000. Cells were collected 72 h after transfection and subjected to Western blotting using references from the literature [9].

### 2.6. SiRNA Interference

Three interference sequences and a negative control were constructed for the target gene in order to ensure the effectiveness of interference. The SiRNA sequence with the best interference efficiency was then assessed against the negative control. Beijing Jereh synthesized these sequences, and the interfering sequences are displayed in Table 2. Before transfection, the interfering group was screened for the most effective sequence.

### 2.7. The Cell Proliferation Activity Assay

Sheep fibroblasts and mouse ovarian fibroblasts were inoculated on 24-well culture plates; the cell confluence in each well was between 40% and 50%. Cells were transfected using Lipofectamine 3000 (Invitrogen; Thermo Fisher Scientific, Waltham, MA, USA) with the overexpression vector pcDNA3.1-MYC-COIL vector plasmid, the pcDNA3.1 empty vector, the interference group, and the negative control group siRNA for 6 h after transfection. The MTT and CCK-8 assay kits were used to study the absorbance of cells transfected for 0, 24, 48, and 72 h.

### 2.8. Detection of the Effect of the Sheep COIL Gene on the Biogenesis Pathway of Spliceosomal U snRNPs

Next, 24-well culture plates were injected with sheep fibroblasts and incubated until the well’s cells reached a confluence of between 40% and 50%. Sheep fibroblasts were co-transfected with the overexpression vector pcDNA3.1-MYC-COIL vector plasmid and the pcDNA3.1 empty vector using Lipofectamine 3000. After a 48 h transfection, the impact of the *COIL* gene overexpression on the spliceosomal U snRNP biogenesis pathway was investigated.

### 2.9. Statistical Methods

All data were analyzed using the one-way measures analysis of variance (ANOVA) and the least significant difference method (LSD) of SPSS 21.0 software. Statistical significance was presented at an α level of *p* < 0.01. Based on SNP analyses, the general linear model (GLM) and mixed linear model (MLM) of the TASSEL 5.0 software (http://www.maizegenetics.net, accessed on 1 November 2023) were used to obtain the correlation values. The GLM uses the group structure information, whereas the MML uses the information of the population structure and the kinship relationship. The fixed effects of the MLM model are parity and population, the random effects are related, and each SNP site is finally associated with a value.

## 3. Results

### 3.1. Polymorphism Analysis of the COIL Gene in Sheep

The results of the polymorphism study revealed that the wild-type genotype frequency was higher at all three *COIL* gene loci in the Hetian sheep population than heterozygous and mutant genotype rates, indicating that both the frequency and the number of mutations were relatively low. According to Table 3, *CC* genotype frequencies at the SNP1 locus were greater in Turpan black sheep and Cele black sheep than *CG* and *GG* genotype frequencies. Although the *CG* phenotype was higher in the Altay and Dolang sheep populations than the *CC* and *GG* phenotypes, suggesting that the SNP1 locus had a higher mutation occurrence in those groups. Additionally, among the SNP3 loci, the *GA* type was more prevalent in the Altay sheep population than the *GG* or *AA* type, indicating that the population had a greater incidence of SNP3 loci mutations.

According to Table 4, in four populations of Altay sheep, Turpan black sheep, Hotan sheep, and Dolang sheep, the *COIL* gene’s SNP1 locus displayed moderate polymorphism information content (PIC) (0.25 < PIC < 0.5). In addition, the heterozygosity of the three loci was high. In two populations of Altay sheep and Hotan sheep, the SNP3 locus displayed moderate polymorphism (0.25 < PIC < 0.5).

### 3.2. Examining the Relationships between Sheep Lambing Features and Polymorphic Genotypes of the COIL Gene

According to Table 5, an association analysis of these three SNPs typing results with lambing traits in sheep revealed that only the genotype of the *COIL* gene COILSNP1 locus was substantially (*p* < 0.05) linked to the mean number of lambs produced in Hetian, Cele Black, and Dorang sheep. The mean lambing number and two *COIL* gene loci, namely, COILSNP2 and COILSNP3, did not substantially correlate (*p* > 0.05).

### 3.3. Tissue Expression Profile Analysis of the COIL Gene in Sheep

**Figure 1:**
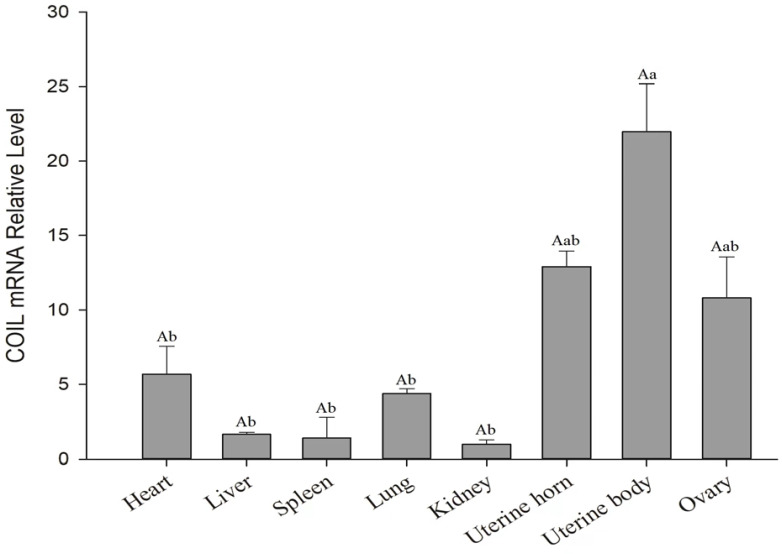
Tissue expression profiling analysis of the sheep *COIL* gene. Different small letter superscripts mean a significant difference (*p* < 0.05); those with different capital letter superscripts mean extremely significant difference (*p* < 0.01); those with the same letter superscripts mean no significant difference (*p* > 0.05).

The expression of the *COIL* gene in various tissues of Hetian multiparous red sheep was analyzed using real-time RT-PCR to further confirm the influence of the *COIL* gene on lambing features and uncover its potential mode of action. According to Figure 1, all eight sites showed a certain degree of *COIL* gene expression although the uterine body had the highest level of *COIL* gene expression. In line with the finding that a *COIL* gene deficiency impacts the development and gamete production, and reduces the number of lambs produced [10], reproductive organs are affected because the *COIL* gene is inevitably expressed in them. The expression was highly significant and considerably higher in the uterine body and uterus than in other tissues, which was in line with the anticipated results.

### 3.4. Effect of Sheep COIL Genes on Cell Proliferation and Activity

**Figure 2:**
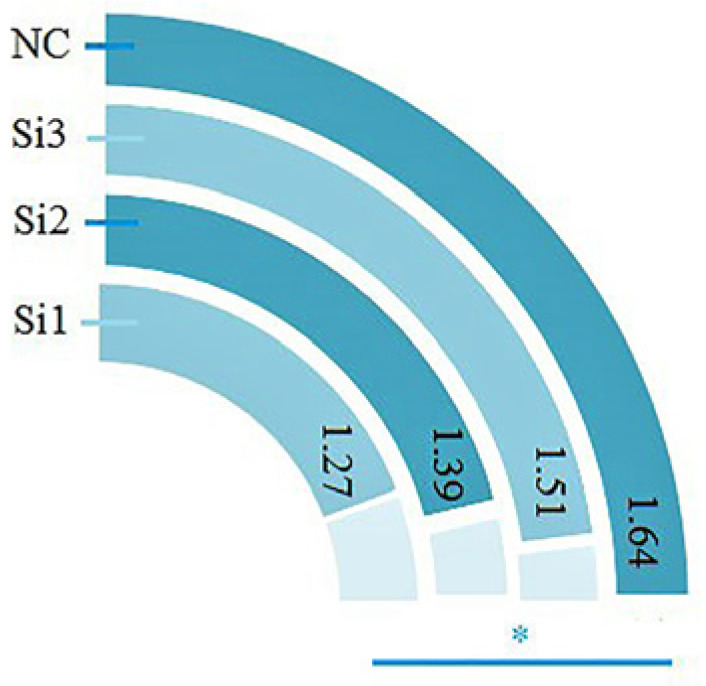
*COIL* interferes with the efficiency of gene siRNA.

The *COIL* gene may be implicated in cell proliferation because it facilitates the biogenesis of spliceosomal U snRNPs. The author created a sheep *COIL* eukaryotic expression vector and a siRNA interference sequence to support this hypothesis. Three siRNA interference sequences were designed; in order to screen for the best interference efficiency, fluorescence quantification was used to measure the interference efficiency after transfection, and Si1 was selected as the interference sequence because it had the best interference efficiency compared with the control group (*p* < 0.5) out of the three interference sequences constructed for the *COIL* gene (Figure 2). 

The effects of *COIL* overexpression and inhibition on cell proliferation and activity were assessed using the CCK-8 and MTT methods after the plasmid pcDNA3.1-Myc-COIL and interfering siRNA were transfected to mouse ovarian fibroblasts and sheep fibroblasts, respectively.

#### 3.4.1. The MTT Cell Survival Assay

**Figure 3:**
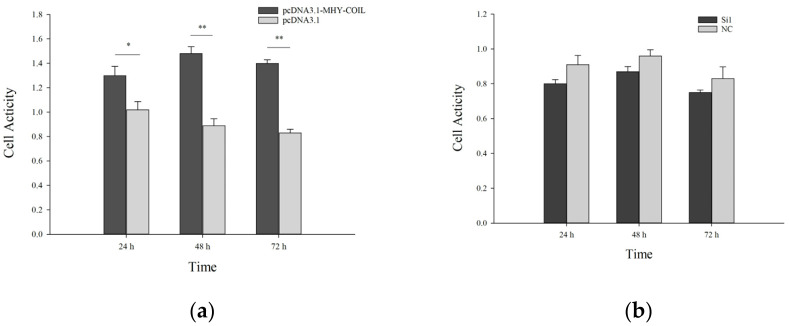
Activity of *COIL* overexpression (**a**) and interference (**b**) in mouse ovarian fibroblasts at various time points. Note: Data are expressed as the means ± SE. Value with * and ** differ significantly at *p* < 0.05 and *p* < 0.01, respectively.

**Figure 4:**
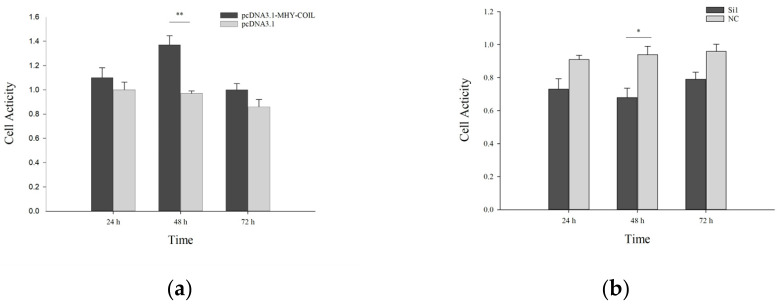
Activity of *COIL* overexpression (**a**) and interference (**b**) in sheep fibroblasts at various time points. Note: Data are expressed as the means ± SE. Value with * and ** differ significantly at *p* < 0.05 and *p* < 0.01, respectively.

According to Figure 3 and Figure 4, the transfection results in mouse ovarian cells and sheep fibroblasts were obtained, and their overall trend of cellular activity was lower than that of the control group after transfection with the interference group. At 48 h after transfection with the overexpression vector, their cellular activity was significantly higher than that of the control group. Although statistically significant levels were reached, the tendency was consistent at subsequent time intervals.

#### 3.4.2. The CCK-8 Cell Proliferation Assay

**Figure 5:**
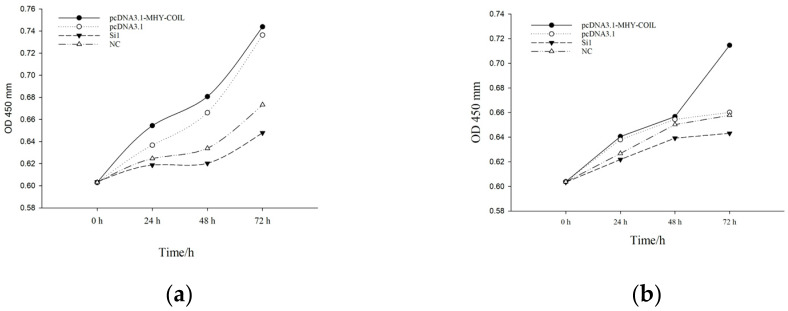
*COIL* overexpression and interference proliferation growth curves of mouse ovarian fibroblasts (**a**) and sheep fibroblasts (**b**).

Figure 5a demonstrates that in mouse ovarian fibroblasts, the number of proliferating cells increased significantly after transfection with the overexpression vector compared with the control group (*p* < 0.05). However, the number of cells that proliferated following transfection with the interfering sequence decreased compared with the control group, and the overall proliferation trend was consistent. Figure 5b demonstrates that at 72 h after being transfected with ovarian fibroblasts, the proliferation of sheep fibroblasts was considerably higher than that of the control group (*p* < 0.05). Similarly, the proliferation of cells in the interfering group was lower than that of the control group.

### 3.5. Detection of the Effect of the Sheep COIL Gene on the Biogenesis Pathway of Spliceosomal U snRNPs

**Figure 6:**
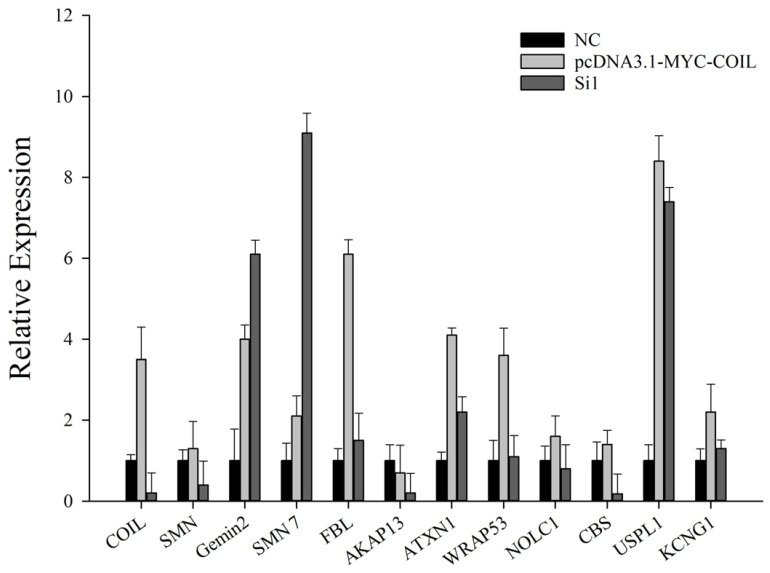
Effect of overexpression and interference with *COIL* on the expression activity of protein-interacting genes.

The biogenesis pathway of spliceosomal U snRNPs is known to be impacted by the *COIL* gene. The sheep ovarian fibroblasts were successfully co-transfected with the overexpression vector pcDNA3.1-MYC-COIL and an interfering siRNA. The effect of the *COIL* gene on their reciprocal genes was examined by RT-PCR to determine whether the *COIL* gene exerted any effect on the activity of genes upstream and downstream of the pathway. Figure 6 displays the outcomes. The overexpression of *COIL* genes considerably elevated the expression of *FBL*, *ATXN1*, *WRAP53*, and *USPL1* genes compared with the control group, as was the expression of *SMN*, *NOLC1*, *CBS*, and *KCNG1* genes. The expression of *SMN7* and *Gemin2* genes, however, was dramatically increased when *COIL* genes interfered with the expression, demonstrating their mutual antagonism with *COIL* genes.

## 4. Discussion

### 4.1. Mechanism of Action of the COIL Gene

We aimed to verify the results of the previous validation, so as to verify the regulatory mechanism regulating the reproductive traits of local sheep breeds in Xinjiang. Using *COIL* knockout mice, Michael et al. examined the impact of *COIL* gene deletion on cell survival and reproductive success [9]. The *COIL* gene (or a tightly connected locus) was knocked out, resulting in the observed hemizygous phenotype, and the *COIL* knockout embryos died at a late gestational stage. Furthermore, *COIL* knockout mice have serious reproductive and fertility issues. Reduced testicular volume was observed in mutant males who survived embryonic lethality, and deletion of 85% of the *COIL* gene’s coding area significantly impacted the formation of the Cajal body in adult tissues and embryonic fibroblasts of mutant mice [11]. Therefore, the *COIL* gene is necessary for the best possible growth and health of the reproductive system.

Recent studies have reported the regulation of genes by constructing eukaryotic expression vectors in sheep cells. Additionally, Wang employed sheep fibroblasts to confirm that sheep oocytes were activated in vitro [12].

We have recently observed a recent increase in studies on the construction and use of eukaryotic expression vectors to control genes in sheep cells. Dong successfully created a eukaryotic expression vector of pEGFP-N1-IGF2, which was transfected into sheep skeletal muscle cells to investigate the mechanism of the *IGF2* gene on myoblast growth and development. Quantitative fluorescence analysis revealed increased expression of *IGF2* [13]. Similarly, Du induced differentiation and immunofluorescence and successfully created a lentiviral expression vector for the sheep *MSTN* gene and concluded that the *MSTN* gene exerted a significant inhibitory effect on the differentiation of fine hair sheep achievement cells [14]. Yu created eukaryotic expression vectors and interference vectors for the *Smo* gene that affects the growth of hair follicles in Tibetan sheep, and the transfection of the best interference vector allowed for the screening of the best interference vector. The mRNA levels were markedly increased (*p* < 0.01), whereas the interference group’s silencing efficacy was higher than 98% [15]. Bovine fetal fibroblasts were transfected with foreign genes via liposomes to achieve the highest transfection efficiency [16]. To investigate the underlying mechanism of the effect of the *COIL* effect on reproductive development, mouse ovarian fibroblasts and sheep fibroblasts were transfected with overexpression vectors and interfering RNA that were created.

### 4.2. Study of the Mechanism of the COIL Gene

We successfully constructed an overexpression vector pcDNA3.1-MYC-COIL, and screened the best-interfering siRNA sequence, which was successfully transfected into mouse ovarian fibroblasts and sheep fibroblasts using Lipofectamine 3000. The overexpression control group significantly increased the mRNA expression of the *COIL* gene in mouse fibroblasts. The *COIL* gene’s mRNA and protein expression were considerably (*p* < 0.01) increased in the overexpression group and significantly (*p* < 0.01) decreased in the interference group.

The developed overexpression vector and an interfering siRNA were transfected into mouse ovarian fibroblasts and sheep fibroblasts to examine the effect of the *COIL* gene on the proliferation of cells [17]. The proliferation rate of both cells was significantly increased following transfection with the overexpression vector at 72 h compared with the control group. However, after transfection with the interfering siRNA, both sheep and mouse ovarian fibroblasts had lower rates of proliferation than the control group. Accordingly, the overexpressed *COIL* gene significantly increased the value-added rate of cells, and promoted the growth and development of reproductive organs, whereas interference with the *COIL* gene reduced the proliferation rate, which affected the development and gamete production [18]. This is consistent with the fact that a deficiency of the *COIL* gene affects the development and production of gametes, affecting the number of lambs produced [5].

Following transfection, we used the CCK-8 kit to measure the activity of cells [19]. In mouse ovarian fibroblasts, the overexpression group’s cell activity was higher than that of the control group at each time point, with a significant effect observed at 48 h. The overall pattern revealed that the reaction time and sample loss were connected to the initial spike in cell activity and the subsequent decrease. Reduced activity was recorded in the interference group than in the control group at each time point. The interference group’s activity was lower than that of the control group’s during each time period, with an overall and relatively stable trend [20]. Cells from the overexpression group in sheep fibroblasts had significantly higher activity than those from the control group at all time points, particularly at 48 and 72 h. The overall pattern was the same; the activity of cells in the interference group was lower than the control group’s activity at each time interval, and it was noticeably lower than the control group’s activity after 48 h [21]. It was hypothesized that the *COIL* gene enhanced the cell activity while activating the regulatory system for cell proliferation, as reflected by the results of the cell activity assay and the trend of the proliferation assay that were in agreement.

### 4.3. Impact of COIL Genes on Spliceosomal U snRNPs’ Biogenesis Pathway

To better understand how *COIL* proteins function, their interacting proteins were predicted using the STRING v10.0 online software [22]. The analysis of protein network interactions revealed that network maps of *COIL* genes interact with their proteins and are linked to Cajal bodies. Small nuclear ribonucleoproteins (snRNPs) are synthesized and recruited by the Cajal body known as *COIL*, and in the absence of *COIL*, the remaining Cajal body loses interaction with the *SMN* complex [23]. Lack of interaction between *SMN* and *COIL* in the nucleus could affect the ability to construct RNPs because *SMN* is required for the assembly and in vivo recycling of snRNPs [24]. This is consistent with experimental findings showing that when the expression of the *COIL* gene is disrupted, the Cajal body loses contact with the *SNM* complex and binds freely, reducing the *SNM* gene expression as well [25]. However, the expression activity of the *SNM7* gene increased extremely significantly, indicating that *SMN* homologous gene expression occurs even in the absence of the Cajal body’s binding [26].

To control p53, a protein that directs RNAs known to be Cajal body specific to Cajal bodies [27]. The *WRAP53* gene produces a p53 antisense transcription product, indicating that the WRAP53 protein is a crucial component in the maintenance of Cajal bodies and that the *SMN* is recruited to Cajal bodies [28]. Thus, the overexpression of the *COIL* gene concurrently increases the expression of the *WRAP53* gene [29]. The overexpressed *COIL* gene binds to more Cajal bodies, indirectly causing the *WRAP53* gene to produce more p53 antisense transcription products, which are used to regulate p53 and maintain response homeostasis.

The *NOLC1* gene aids in the shuttling of proteins between the nucleolus and cytoplasm by binding to the nucleolus and a Cajal body phosphoprotein, a nuclear localization signal-binding protein [30]. The expression of the *NOLC1* gene is simultaneously increased and interference is decreased when *COIL* genes are overexpressed [31]. To study the relationships between the remaining genes and the *COIL* gene overexpression and interference, the remaining genes were also quantified for the first time.

The *COIL* gene has been reported to affect the spliceosomal U snRNP biogenesis pathway [32], which primarily regulates reproductive systems by transporting and localizing its protein translation process.

## 5. Conclusions

Three SNPs were identified on sheep chromosome XI of the *COIL* gene. The association analysis demonstrated that COILSNP1 was strongly correlated with litter size. The cell proliferation and cell activity assays revealed that the *COIL* gene promoted the biogenesis synthesis of spliceosomal U snRNPs and dramatically increased the proliferation and activity of mouse ovarian fibroblasts and sheep fibroblasts. These findings imply that *COIL* may be involved in the regulation of the features related to lambing number in sheep.

## Figures and Tables

**Table 1:** Information of SNPs.

Gene Name	SNP Site	Position	Primer Sequence
*COIL*	COILSNP1	7321466	F1: GAAGGTCGGAGTCAACGGATTCCATGAAAGAACCTGGGAAA F2: GAAGGTGACCAAGTTCATGCTCCATGAAAGAACCTGGGAAC R1: CCTCAGCTCCATTTTCGTTG
*COIL*	COILSNP2	7314134	F1: GAAGGTCGGAGTCAACGGATTGACTCCGAGGAGGAATCGC F2: GAAGGTGACCAAGTTCATGCTGACTCCGAGGAGGAATCGG R1: GTGGCATGGTCGTCCGTAC
*COIL*	COILSNP3	7321563	F1:GAAGGTCGGAGTCAACGGATTGCACAGTCTGTGAAAGAGTGGA F2: GAAGGTGACCAAGTTCATGCTGCACAGTCTGTGAAAGAGTGGG R1: TCTAGCAGGAAGAGCTTTAGGG

**Table 2:** Primer sequence of the target gene.

Gene	Sequence (5′-3′)	Length (bp)
*COIL*	Si1	CCAUCAUCACAGGCUCCAATT
	Si2	CCAGAAGUGCAGCCCUAAATT
	Si3	GCAAAGAAGCGGGCAUUUATT

**Table 3:** Allele and genotype frequencies of identified SNPs of the *COIL* gene in different sheep breeds.

SNP Site	Breed	Number	Genotype Frequency			Allele Frequency	
			*CC*	*CG*	*GG*	*C*	*G*
COILSNP1	Altay sheep	65	0.450	0.483	0.067	0.692	0.308
	Turpan black sheep	240	0.570	0.338	0.092	0.739	0.261
	Hetian duotai hong sheep	62	0.580	0.360	0.060	0.760	0.240
	Cele black sheep	44	0.720	0.260	0.020	0.850	0.150
	Duolang sheep	16	0.190	0.560	0.250	0.470	0.530
			*AA*	*AC*	*CC*	*A*	*C*
COILSNP2	Altay sheep	65	/	/	/	/	/
	Turpan black sheep	240	/	/	/	/	/
	Hetian duotai hong sheep	62	0.990	/	0.010	0.990	0.010
	Cele black sheep	44	/	/	/	/	/
	Duolang sheep	16	/	/	/	/	/
			*GG*	*GA*	*AA*	*G*	*A*
COILSNP3	Altay sheep	65	0.323	0.677	/	0.662	0.338
	Turpan black sheep	240	/	/	/	/	/
	Hetian duotai hong sheep	62	/	0.530	0.470	0.263	0.737
	Cele black sheep	44	/	/	/	/	/
	Duolang sheep	16	/	/	/	/	/

Note: / stands for genotype failure or undiscovered genotype.

**Table 4:** Population genetic analysis of three loci of the *COIL* gene in different sheep breeds.

SNP Site	Breed	PIC	Heterozygosity	Ne
COILSNP1	Altay sheep	0.336	0.427	1.744
	Turpan black sheep	0.311	0.386	1.628
	Hetian duotai hong sheep	0.297	0.363	1.569
	Cele black sheep	0.224	0.257	0.345
	Duolang sheep	0.374	0.498	1.992
COILSNP2	Hetian duotai hong sheep	0.020	0.020	1.020
COILSNP3	Altay sheep	0.347	0.446	1.806
	Hetian duotai hong sheep	0.312	0.387	1.632

Note: PIC, polymorphism information content; Ne, the number of effective allele.

**Table 5:** Correlation analysis between different COILSNP1 genotypes and litter size.

Population	Trait	Genotype		
		*CC*	*CG*	*GG*
Hetian duotai hong sheep	Average number of lambs	1.60 ± 0.55 ^a^	1.39 ± 0.54 ^ab^	1.17 ± 0.40 ^b^
Cele black sheep	Average number of lambs	1.65 ± 0.48 ^a^	1.39 ± 0.49 ^b^	1.50 ± 0.71 ^ab^
Duolang sheep	Average number of lambs	1.15 ± 0.37 ^b^	1.35 ± 0.49 ^b^	1.80 ± 0.45 ^a^

^a, b^ Within a row, means sharing different superscript letters differ significantly (*p* < 0.05).

## Data Availability

The data used in this study are the property of the College of Animal Science, Xinjiang Agricultural University and are therefore not publicly available.
